# An Epidermal Sweat Patch for Tracking Metabolite Levels and Correlation Analysis in Biofluids

**DOI:** 10.1002/advs.78544

**Published:** 2026-10-29

**Authors:** Yao Chen, Xinhao Wan, Haoxin Fan, Hengwei Lou, Jialu Wang, Jie Gao, Dan Wen

**Affiliations:** ^1^ Department of Hepatobiliary Surgery and Liver Transplantation The Second Affiliated Hospital of Xi'an Jiaotong University Xi'an People's Republic of China; ^2^ State Key Laboratory of Solidification Processing School of Materials Science and Engineering Northwestern Polytechnical University Xi'an People's Republic of China; ^3^ School of Life Sciences Northwestern Polytechnical University Xi'an People's Republic of China

**Keywords:** biofluids, correlation analysis, glucose monitoring, metal aerogels, wearable biosensors

## Abstract

Continuous, in situ monitoring of sweat biomarkers offers a promising approach for non‐invasive metabolic assessment, but its practical implementation remains limited by sensing stability and the insufficiently defined physiological relationship between sweat and systemic biofluids. Here, we develop an epidermal sweat patch that integrates a non‐enzymatic electrochemical interface based on Au–Ni(OH)_2_ dual aerogels for glucose monitoring. The complementary roles of Ni(OH)_2_‐mediated glucose adsorption and Au‐assisted electrooxidation enable sensitive and stable sensing, with a calculated detection limit of 0.1 µM and >95% signal retention after 5 weeks. Combined with microfluidic sweat sampling, signal processing and wireless communication, the patch enables real‐time on‐body tracking of sweat glucose during daily activities. In human studies, sweat glucose shows positive associations with interstitial fluid and blood glucose, with Pearson correlation coefficients of up to 0.828, while subgroup analyses further reveal interindividual variability. These results support the physiological relevance of sweat glucose as a complementary signal for metabolic monitoring. Overall, this work integrates non‐enzymatic sensing, wearable system design and cross‐biofluid analysis within a single platform, providing a practical framework for physiologically interpretable sweat‐based glucose monitoring.

## Graphical abstract

This work presents an epidermal sweat patch for continuous glucose monitoring that validates a strong correlation between sweat, interstitial fluid and blood, enabling a reliable and non‐invasive platform for systemic health management.

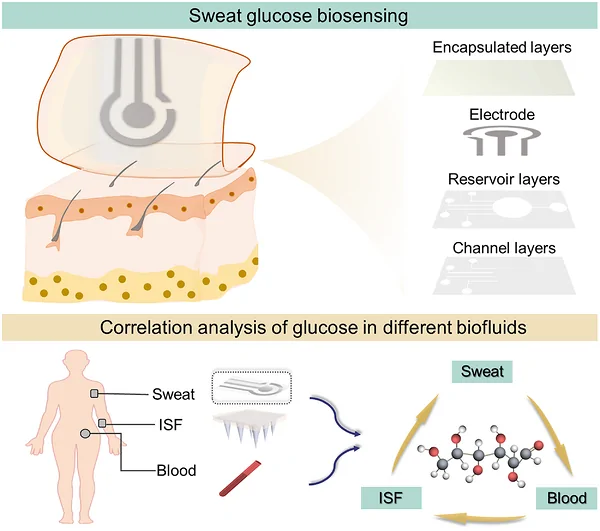

## 1 Introduction

Metabolic diseases arising from abnormalities in metabolite regulation, such as obesity, cardiovascular disorders, and hypertension, have emerged as major global public health concerns [1]. Metabolites, typically small molecules with molecular weights below 1500 Da, serve as key biochemical markers that undergo dynamic transformations during metabolism [2, 3, 4]. Fluctuations in metabolites reflect the real‐time biochemical state of the body and influence health throughout life, from infancy to old age [1, 5, 6, 7]. Routine monitoring of metabolites can substantially reduce the risk and progression of metabolic disorders, including diabetes, gout, and hyperlipidemia [8]. However, conventional metabolite analysis relies on blood sampling followed by benchtop quantification under professional supervision [9, 10, 11, 12]. These approaches are inherently invasive and limited to single time‐point measurements, precluding real‐time or continuous assessment. Therefore, there is an urgent need to develop portable, non‐invasive technologies capable of continuous metabolite monitoring to support personalized health management and improve the prediction and prevention of metabolism‐related diseases [13, 14, 15].

With advances in wearable electronics, wearable biosensors have emerged as powerful tools for continuous, longitudinal health monitoring, offering seamless integration with the human body without discomfort or disruption to daily activities. These devices enable real‐time tracking of physiological and metabolic status across diverse populations, from healthy individuals to patients with chronic diseases [6, 16, 17, 18, 19, 20]. Particularly, skin‐interfaced wearable sweat biosensors (WSBs) have gained substantial attention for their ability to non‐invasively detect a wide range of metabolites in sweat. Rapid progress in this field has revealed considerable potential for disease diagnosis, personalized therapy, fitness assessment, drug monitoring, and large‐scale clinical studies [9, 21, 22, 23, 24, 25, 26, 27, 28].

Despite these advances, the widespread implementation of WSBs remains limited by the predominant use of enzyme‐based sensing strategies. Enzymatic sensors are highly susceptible to environmental variations including pH, temperature, and ionic strength, which can compromise sensitivity, reduce operational stability, and shorten device lifespan under real‐world conditions [29]. These intrinsic limitations hinder the practical translation into clinical and daily‐use scenarios. Non‐enzymatic sensing, which leverages robust catalytic interfaces instead of fragile biological components, offers a promising alternative. The rational design of high‐performance non‐enzymatic sensing interfaces tailored to the molecular structures of specific metabolites thus represents a critical pathway toward the deployment of WSBs for continuous, non‐invasive health monitoring.

Beyond sensing performance, clinical translation is further hindered by limited validation of biomarker correlations between sweat and other biofluids, particularly interstitial fluid (ISF) and blood. Unlike blood‐based diagnostics that are well‐established in clinical practice, sweat analysis has not yet achieved comparable acceptance due to uncertainties regarding its relevance to systemic biomarkers. Although sweat contains abundant biochemical information, the extent to which metabolite concentrations in sweat reflect those in ISF or blood remains insufficiently defined [30, 31, 32, 33, 34, 35]. A comprehensive understanding of metabolite levels across these biofluids, along with the establishment of quantitative correlations among them, is essential for the broad clinical adoption, commercialization, and large‐scale implementation of WSB technologies.

To address these challenges, we develop an integrated epidermal sweat patch for non‐invasive and personalized monitoring of metabolic status, using glucose as a representative biomarker for diabetes, the most prevalent metabolic disorder [36, 37]. The device integrates a non‐enzymatic metal aerogel‐based sensing chip with a microfluidic module and wireless circuitry for real‐time sweat glucose analysis. The rationally designed Au‐Ni(OH)_2_ dual aerogel provides abundant catalytic and adsorption sites, enabling a detection limit of 0.1 µM, a broad linear range (1 µM–3 mM), and exceptional long‐term stability (>95% signal retention over 5 weeks). Experimental results supported by theoretical calculations suggest that synergistic interactions between Au and Ni(OH)_2_ optimize glucose adsorption and electrooxidation, resulting in markedly enhanced sensing performance. In on‐body trials, the sweat patch enables real‐time and accurate monitoring of glucose dynamics, with positive correlations observed in ten participants (five healthy and five with diabetes) under a standardized postprandial protocol. Rather than pursuing analytical sensitivity alone, this work integrates a robust non‐enzymatic sensing interface, microfluidic sweat handling, wireless readout, and cross‐biofluid physiological correlation analysis within a single wearable platform. By linking sweat glucose dynamics with corresponding changes in ISF and blood, the platform extends wearable sweat sensing from isolated analyte detection toward physiologically interpretable metabolic monitoring, providing a practical framework for the future translation of sweat‐based biosensing.

## 2 Results and Discussion

### 2.1 Design and Overview of the Epidermal Sweat Patch

The epidermal sweat patch consists of a microfluidic chip and an Au‐Ni(OH)_2_‐based electrochemical sensing chip, externally connected to a circuit board and Bluetooth module for signal conditioning and wireless transmission (Figure 1). The microfluidic layer, fabricated via polydimethylsiloxane (PDMS) molding, incorporates four inlets, one outlet, and a reservoir that efficiently collects, transports, and stores sweat prior to analysis by the sensing array. At the core of the device is the Au‐Ni(OH)_2_ aerogel‐based electrochemical interface for non‐enzymatic glucose detection. Its porous dual‐aerogel architecture provides a large electroactive surface area and enhances catalytic activity toward glucose oxidation. This non‐enzymatic strategy offers superior sensitivity and stability compared with traditional enzymatic sensors, making it well suited for continuous sweat glucose monitoring. Through the integration of microfluidic sweat harvesting, a high‐performance electrochemical sensor, and wireless electronics, the patch provides a robust platform for non‐invasive, continuous metabolite monitoring and biofluid correlation analysis.

**FIGURE 1:**
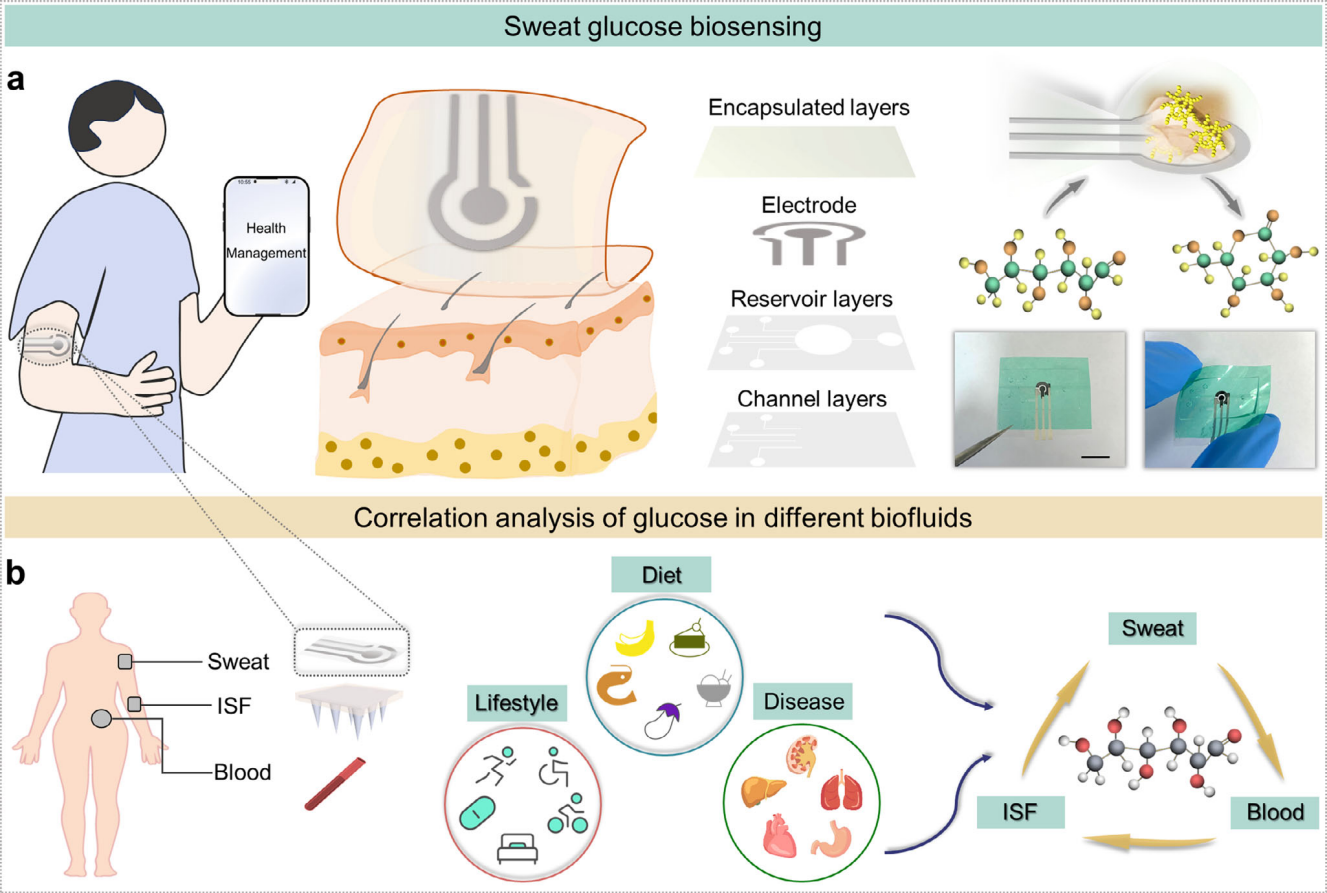
An epidermal sweat patch for health management. (a) Schematic illustration of the wearable sweat patch for non‐invasive glucose biosensing, (b) Conceptual diagram depicting the correlation of glucose levels among human sweat, ISF, and blood.

### 2.2 Non‐Enzymatic Sensing Materials Design and Synthesis

Electrochemical sensing is well suited for wearable applications owing to its portability, compatibility with continuous monitoring, and ease of miniaturization [38]. Glucose, an electroactive monosaccharide, undergoes electrocatalytic oxidation reactions that can be greatly enhanced by rational interface engineering. Noble metals such as Au provide abundant catalytically active sites that facilitate glucose dehydrogenation and electron transfer during electrooxidation [33]. In parallel, transition‐metal hydroxides, such as Ni‐based hydroxides offer abundant adsorption sites that strongly coordinate with glucose molecules [39]. Integrating these two components creates a synergistic catalytic interface. To further amplify this synergy, metal aerogels were employed as the structural scaffold. Their three‐dimensional (3D) interconnected networks provide high surface area, hierarchical porosity, and continuous conductive pathways, which together promote mass and electron transport and increase the accessibility of active sites [40]. Guided by this design rationale, Au‐Ni(OH)_2_ dual aerogels were synthesized through a one‐step gelation process, where pH adjustment converted Ni^2+^ to Ni(OH)_2_ (Figure 2a).

**FIGURE 2:**
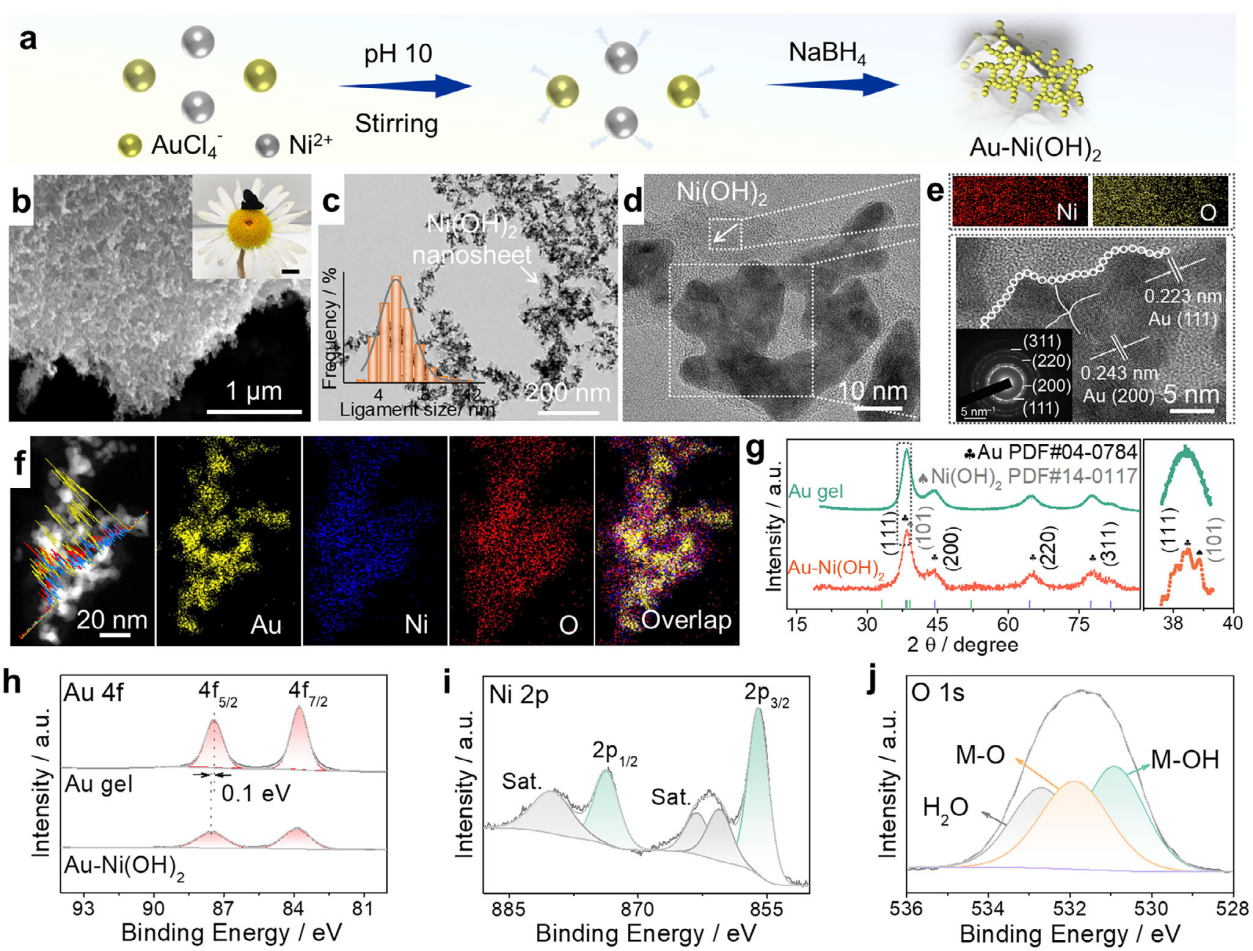
Synthesis and characterization of Au‐Ni(OH)_2_ aerogels. (a) Schematic illustration of the formation process of Au‐Ni(OH)_2_ aerogels. (b–f) Morphological characterization: SEM (b), TEM (c), HRTEM and elemental mapping (d, e), high‐angle annular dark‐field scanning transmission electron microscopy with corresponding elemental mapping (f). Insets in (b), (c), and (e) show a digital photograph of Au‐Ni(OH)_2_ aerogels floating on a flower (scale bar: 1 cm), the nanowire size distribution histogram, and the SAED pattern, respectively. (g) XRD pattern of Au‐Ni(OH)_2_ aerogels. (h–j) High‐resolution XPS spectra of Au 4f (h), Ni 2p (i), and O 1s (j).

The morphology and structure of the resulting Au‐Ni(OH)_2_ aerogels were characterized by scanning electron microscopy (SEM) and transmission electron microscopy (TEM). SEM images show networks of 1D branched nanochains network anchored onto 2D nanosheets (Figure 2b). TEM analysis further confirms that the aerogels consist of interconnected Au nanochains (average ligament size ≈ 5.7 nm) intertwined with Ni(OH)_2_ nanosheets tens of nanometers in size (Figure 2c,d). High‐resolution TEM (HRTEM) reveals Au (111) and (200) lattice spacings (0.223 and 0.243 nm) and numerous grain boundaries (Figure 2e), which are known to enhance electrocatalytic activity [41]. Selected‐area electron diffraction (SAED) exhibits Au diffraction rings, while the absence of observable Ni(OH)_2_ lattice fringes suggests its amorphous nature (inset Figure 2e). Elemental mapping indicates uniform distribution of Au, Ni, and O throughout the aerogel network, forming a core–shell‐like configuration, with Au‐rich cores and Ni‐enriched shells (Figure 2f and Figure S1a). TEM‐energy‐dispersive X‐ray spectroscopy analysis yields an Au/Ni atomic ratio of 19.0/14.2 (Figure S1b).

Distinct X‐ray diffraction (XRD) peaks at 38.1^°^, 44.4^°^, 64.6^°^ and 77.5^°^ correspond to the (111), (200), (220) and (311) planes of face‐centered cubic (*fcc*) Au (JCPDS#04‐0784), consistent with SAED results (Figure 2g). A weak peak at 38.5° in the magnified XRD pattern is attributed to the (101) plane of amorphous Ni(OH)_2_ (JCPDS#14‐0117), confirming the coexistence of metallic Au and Ni(OH)_2_ phases. The X‐ray photoelectron spectroscopy (XPS) survey spectrum (Figure S2) confirms the presence of Au, Ni, O, and C elements. The high‐resolution Au 4f spectrum shows Au primarily in the metallic state, with a slight positive binding energy shift of 0.1 eV relative to pure Au gel, likely arising from electronic interactions with Ni (Figure 2h) [42]. The Ni 2p features Ni^2+^ at 856.0 and 873.8 eV and their associated satellite peaks at 861.4 and 880.1 eV (Figure 2i). The O 1s spectrum contains three components at 530.9, 531.9, and 532.7 eV, attributable to M─OH, M─O, and adsorbed H_2_O species, respectively (Figure 2j).

Collectively, these structural and compositional analyses validate the successful formation of Au‐Ni(OH)_2_ dual aerogels. The integration of 1D Au nanochains and 2D Ni(OH)_2_ nanosheets endows the aerogel framework with abundant reactive sites, enhanced molecular adsorption, and strong catalytic coupling, synergistic features that promote efficient electrocatalysis and ultimately boost glucose sensing performance [43].

### 2.3 Glucose Electrocatalysis and Sensing

The glucose electrocatalytic reaction (GER) was first evaluated by cyclic voltammetry (CV) in 0.1 M phosphate‐buffered saline (PBS, pH 7.4) (Figure 3a,b and Figure S3). Upon the addition of 2 mM glucose, the Au‐Ni(OH)_2_ modified glassy carbon electrode (Au‐Ni(OH)_2_/GCE) shows a pronounced increase in oxidation current, while control electrodes show negligible changes. Notably, the GER response current at 0.35 V (vs. Ag/AgCl) reaches 14.7 µA on Au‐Ni(OH)_2_/GCE, which is 5.89‐ and 45.9‐fold higher than those of Au gel/GCE and Ni(OH)_2_/GCE, respectively, demonstrating the superior catalytic activity of Au‐Ni(OH)_2_. The electrochemical active surface areas (ECSAs) of Au‐Ni(OH)_2_/GCE was estimated to be 0.45 cm^2^ according to the Randles‐Sevick equation [44], approximately 1.15‐ and 1.45‐fold larger than those of Au gel/GCE and Ni(OH)_2_/GCE, indicating a larger electrochemically active surface area (Figure S4). To further probe glucose adsorption, open‐circuit potential (OCP) measurements, which reflect variations in the Helmholtz layer, were performed [45]. As shown in Figure 3c, the OCP drops dramatically upon glucose addition. The potential change on Ni(OH)_2_/GCE (0.067 V) is 9.57 and 4.18 times higher than those on Au gel/GCE and Au‐Ni(OH)_2_/GCE, respectively, indicating the strongest glucose adsorption on Ni(OH)_2_/GCE. These results suggest that Au sites primarily catalyze glucose electrooxidation, whereas Ni(OH)_2_ contributes to glucose adsorption, and that their synergistic interaction promotes the GER.

**FIGURE 3:**
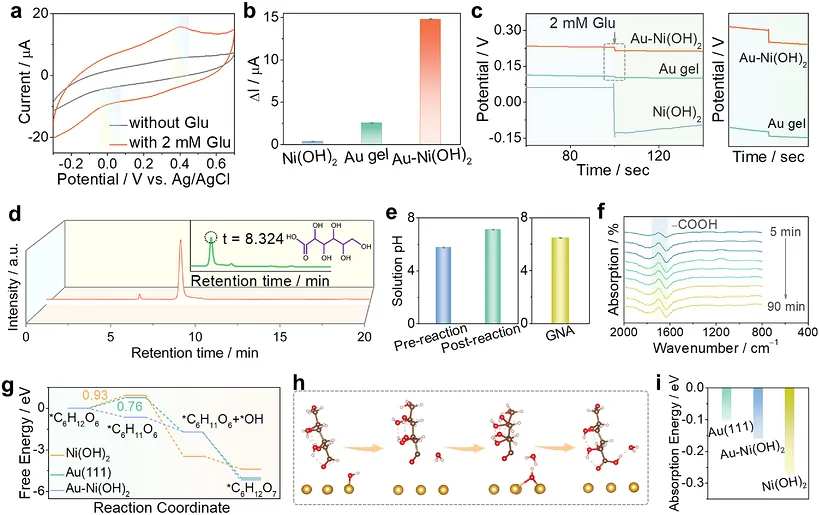
Glucose electrocatalysis on Au‐Ni(OH)_2_ aerogels. (a) CV curves of Au‐Ni(OH)_2_/GCE for glucose oxidation in 0.1 M PBS (pH 7.4). (b) Comparison of glucose oxidation currents on different modified GCEs. (c) OCP curves of Au, Ni(OH)_2,_ and Au‐Ni(OH)_2_ aerogels, respectively. (d) HPLC chromatograms of glucose oxidation products after continuous testing. Inset shows the chromatograms of pure gluconic acid. (e) Histogram of solution pH before and after successive tests, alongside the pH of pure gluconic acid. (f) In situ FT‐IR spectra of glucose oxidation at various reaction times. (g) Calculated Gibbs free‐energy profiles for the proposed glucose oxidation pathway on Au(111), Ni(OH)_2_, and Au–Ni(OH)_2_ surfaces. (h) Schematic illustration of the proposed glucose oxidation pathway on the Au–Ni(OH)_2_ interface. (i) Adsorption energies of glucose on Au(111), Ni(OH)_2_ and Au‐Ni(OH)_2_, respectively. Note: glucose is abbreviated as Glu here.

High‐performance liquid chromatography (HPLC) and in situ Fourier transform infrared spectroscopy (FT‐IR) were conducted to analyze the electrochemical oxidation products. A 5 mM glucose solution was electrolyzed for 12 h at 0.1 V in 0.1 M PBS (pH 7.4) prior to HPLC analysis. As illustrated in Figure 3d, a product peak at 8.312 min matched the retention time of standard gluconic acid (8.324 min), confirming that gluconic acid is the primary oxidation product. This suggests a two‐electrons GER under mild conditions, coinciding with previous reports [46, 47]. Additionally, the solution pH decreased from 7.07 to 6.48, approaching that of standard gluconic acid, further corroborates its formation (Figure 3e). In situ FT‐IR spectra collected at 0.1 V (Figure 3f) show a broad increasing band at ∼1693 cm^−1^, assigned to HCOO^*^ adsorption [48]. The intensity of this band increased over 5–90 min, indicating the accumulation of reaction products on the Au‐Ni(OH)_2_ surface. No additional characteristic bands were detected, suggesting that gluconic acid is the predominant product detected under the tested conditions.

Based on these experimental findings, density functional theory (DFT) calculations were performed to further examine the glucose oxidation pathway on Ni(OH)_2_, Au(111), and Au–Ni(OH)_2_ surfaces (Figure S5). The calculated Gibbs free‐energy profiles suggest that glucose (C_6_H_12_O_6_) first adsorbs on the catalytic surface to form ^*^C_6_H_12_O_6_, followed by dehydrogenation to ^*^C_6_H_11_O_6_ and subsequent OH‐assisted oxidation to ^*^C_6_H_12_O_7_, which is finally released as gluconic acid (Figure 3g,h). Thus, the proposed pathway is C_6_H_12_O_6_→^*^C_6_H_12_O_6_→^*^C_6_H_11_O_6_→^*^C_6_H_11_O_6_+^*^OH→^*^C_6_H_12_O_7_→C_6_H_12_O_7_. The dehydrogenation step was considered the rate‐determining step, consistent with previous mechanistic studies of glucose electrooxidation on Au under phosphate‐buffered conditions [49, 50]. For this step, the calculated reaction free‐energy changes are +0.93 eV on Ni(OH)_2_ and +0.76 eV on Au(111), whereas the corresponding transformation becomes thermodynamically favorable on the Au─Ni(OH)_2_ interface (ΔG < 0). This comparison indicates that the Au─Ni(OH)_2_ interface substantially lowers the thermodynamic free‐energy requirement for glucose dehydrogenation. These ΔG values represent reaction free‐energy changes between intermediates and are therefore used here to compare the relative thermodynamic favorability of the dehydrogenation step on different catalyst surfaces, rather than as explicit transition‐state activation barriers. In addition, Ni(OH)_2_ exhibits the strongest glucose adsorption energy (−0.27 eV), compared with Au–Ni(OH)_2_ (−0.16 eV) and Au(111) (−0.10 eV), consistent with the OCP results (Figure 3i). Together, these results suggest that Ni(OH)_2_ primarily promotes glucose adsorption, while the Au‐containing interface facilitates the subsequent dehydrogenation and oxidation processes, thereby accounting for the enhanced glucose electrocatalytic activity of the Au–Ni(OH)_2_ dual aerogel.

The remarkable electrocatalytic activity of Au‐Ni(OH)_2_ toward glucose under neutral conditions demonstrates its feasibility as a high‐performance non‐enzymatic glucose sensing interface. A working potential of 0.1 V was selected to balance sensitivity and selectivity (Figure S6). Current‐time (i‐t) curves (Figure 4a) show stepwise increases in current upon successive glucose additions. As shown in Figure 4b, two distinct linear relationships are observed, 1–200 µM (R_1_^2^ = 0.996) and 200 µM–3 mM (R_2_^2^ = 0.993), respectively, covering the typical glucose concentration range in sweat. The limit of detection (LOD) was estimated to be 0.1 µM (LOD = 3σ/S, where σ represents the standard deviation of the blank signal and S represents the slope of the calibration curve in the low‐concentration region). Such a high sensitivity response is attributed to the synergistic catalytic interplay between Au and Ni(OH)_2_, which facilitates rapid glucose adsorption and improves thermodynamic favorability.

**FIGURE 4:**
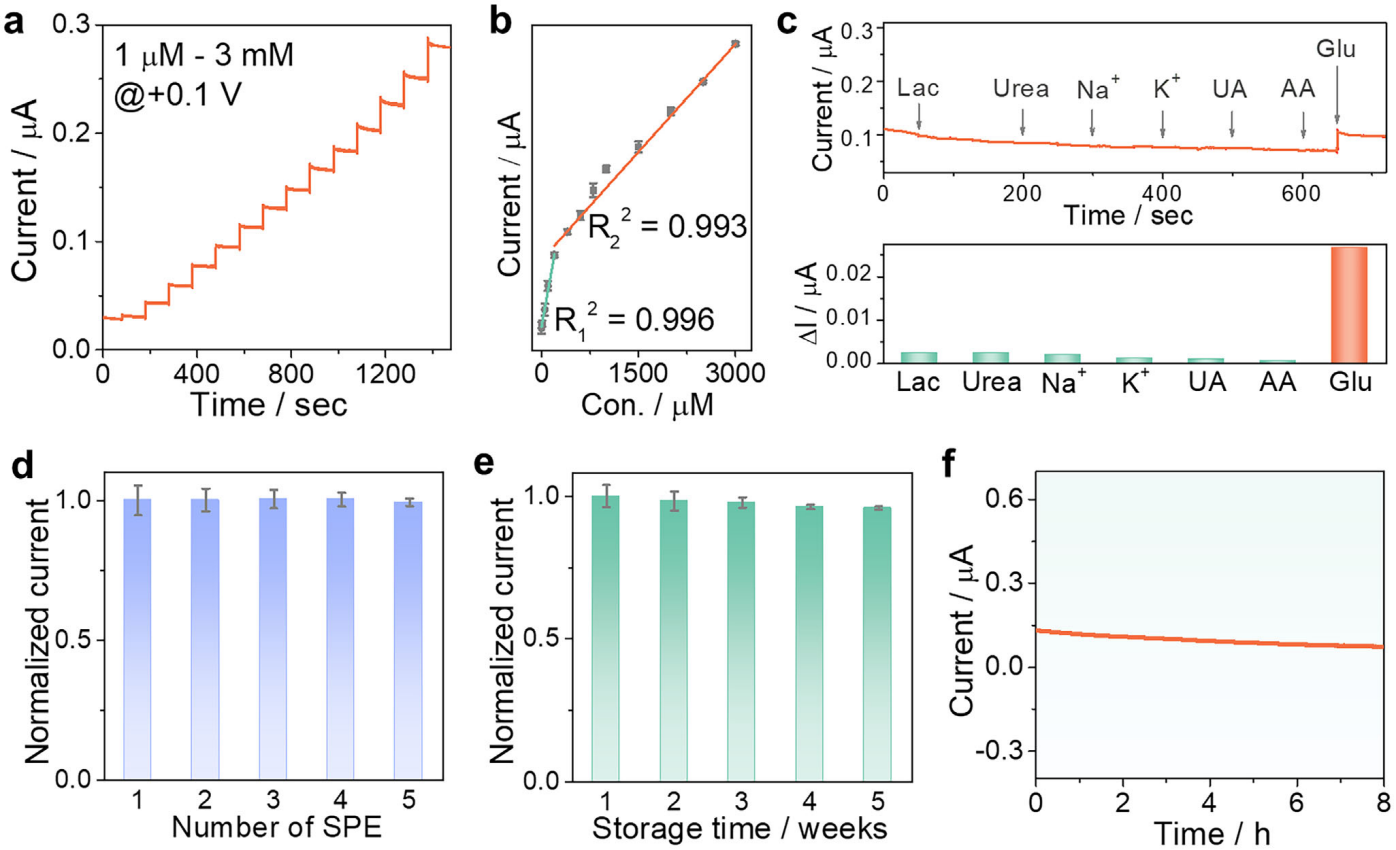
Performance of the non‐enzymatic glucose sensor based on Au‐Ni(OH)_2_ aerogels. (a) I‐t curves and (b) the corresponding calibration plot of Au‐Ni(OH)_2_/SPE in 0.1 M PBS (pH 7.4) at 0.1 V (vs. Ag/AgCl). (c) I‐t curve and histograms of the current responses from anti‐interference tests. Interferent concentrations: 5 mM of lactate (Lac), 1 mM of Urea, 5 mM of NaCl and KCl, 100 µM of uric acid (UA), and 50 µM of ascorbic acid (AA), respectively. (d) Histograms of current responses across different SPEs and (e) for the same SPE under various storage times. (f) I‐t curve of Au‐Ni(OH)_2_/SPE in 0.1 M PBS (pH 7.4) containing 50 µM glucose during continuous testing.

The selectivity, reproducibility, and stability of the Au─Ni(OH)_2_‐based biosensor were subsequently assessed. As shown in Figure 4c, the sensor exhibits negligible responses to high concentrations of common interferents, indicating excellent selectivity. The reason for choosing these interferents is that they represent physiologically relevant levels of major electroactive or abundant constituents encountered in human sweat [28]. The relative standard deviation (RSD) of current responses across five independently fabricated electrodes is 4.79%, and the sensor retains 95.8% of its initial response after 5 weeks of storage (Figure 4d,e), demonstrating good reproducibility and long‐term stability. Additionally, after continuous operation for 8 h, the current response decreases by only 11.2% (Figure 4f). This durability benefits from the non‐enzymatic sensing interface, which avoids enzyme degradation while maintaining reliable operation over relevant environmental conditions. Furthermore, the cross‐linked nano‐network structure of the metal aerogels provides mechanical robustness and suppresses aggregation or structural degradation during electrochemical operation.

The environmental robustness of the Au–Ni(OH)_2_‐based glucose sensor was further evaluated under different pH and temperature conditions (Figure S7). The normalized current response varied by less than 9.04% across the investigated pH range and by less than 10.1% over 25–40°C, indicating limited environmental dependence under conditions relevant to wearable sweat monitoring. These results, together with the absence of enzyme‐related degradation, further support the robustness of the non‐enzymatic sensing interface for on‐body applications.

Notably, the Au─Ni(OH)_2_ aerogel‐based biosensor shows competitive analytical performance compared with representative reported sensors (Table S1), owing to its efficient electrocatalysis toward glucose oxidation under neutral conditions. Beyond PBS, the biosensor also demonstrates robust performance in artificial sweat, ISF, and human serum, underscoring its applicability in diverse biofluids (Figures S8–S10). Particularly, in artificial sweat, the Au‐Ni(OH)_2_ modified screen printed electrode (SPE) exhibits two well‐defined linear ranges, 1–100 µM (R_1_^2^  =  0.998) and 100 µM–3 mM (R_2_^2^  =  0.990), fully covering the physiologically relevant glucose levels in sweat. These results establish the feasibility of Au─Ni(OH)_2_ for reliable glucose detection in real‐sample validation and wearable applications.

### 2.4 Wearable Application

Microfluidic platforms enable efficient, low‐volume sweat collection and transport, minimizing evaporation and contamination for real‐time analysis. Here, we designed a microfluidic chip featuring four inlets, a sensing reservoir, and an evaporation outlet (Figure 5a and Figures S11–S14). Numerical simulations using COMSOL Multiphysics were conducted to visualize and analyze sweat flow within the device (Figure 5b and Figures S15–S17). The simulations show that the chip achieves high temporal resolution in liquid collection across a range of sweat flow rates, confirming its strong potential for precise microfluidic sampling and delivery. On‐body testing further reveals flow behaviors consistent with the simulated results, validating the reliability of the chip design under physiological conditions.

**FIGURE 5:**
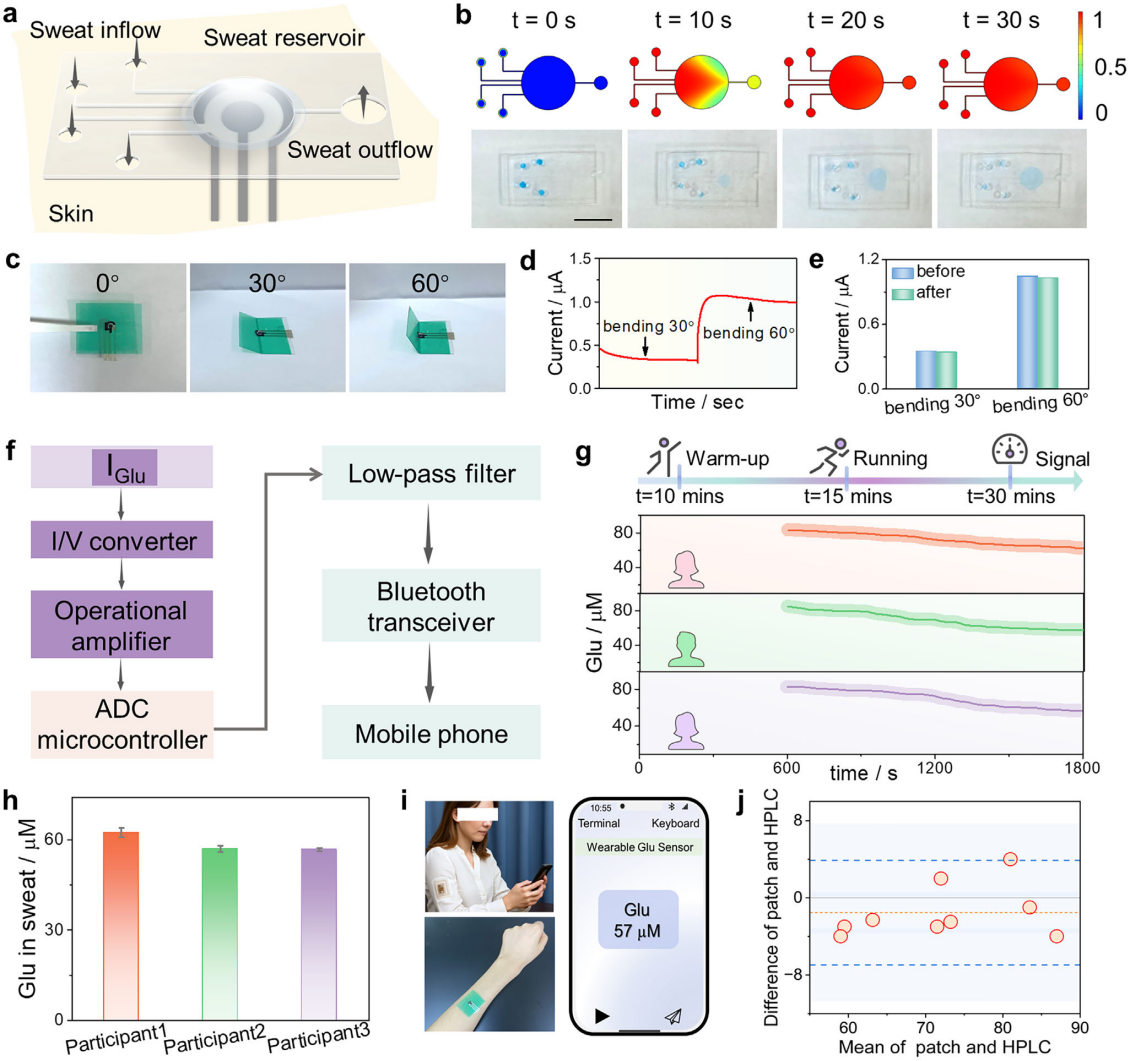
On‐body evaluation of the epidermal sweat patch. (a) Schematic illustration of the microfluidic chip for sweat collection. (b) Comparison of theoretical simulations and experimental measurements of sweat flow in the microfluidic chip at 4 µL min^−1^. (c) Optical images of SPEs under different bending angles, (d) the corresponding i‐t curves, and (e) the relationship between current responses and bending angles. (f) Block diagram of the integrated circuit system. (g, h) Real‐time sweat glucose monitoring in three participants during exercise. (i) Optical images of a female participant wearing the epidermal sweat patch during on‐body testing. (j) Bland–Altman analysis comparing sweat glucose concentrations measured by the wearable sensor and HPLC using nine paired measurements obtained from three participants at 10, 20, and 30 min during exercise.

Mechanical deformation, particularly bending, frequently occurs during daily human activities, thus, mechanical flexibility is essential to ensure stable and reliable signal readouts from the wearable sweat patch. Prior to on‐body evaluation, bending tests were performed at different angles (Figure 5c–e). The change in response current, defined as ΔI = I_0_‒I^′^ (where I_0_ and I^′^ represent the peak currents under flat and bent conditions, respectively), remains negligible at bending angles of 30° and 60°, with ΔI < 1.0 nA during glucose detection. This mechanical robustness arises from the highly interconnected network of the metal aerogel, which resists structural deformation and maintains consistent electrocatalytic performance.

On‐body evaluation for real‐time, in situ sweat glucose monitoring was implemented on three healthy participants (two females and one male). Sweat generated during exercise was continuously collected by the microfluidic chip and delivered to the sensing region, where glucose levels were monitored in real time using a pre‐established calibration curve (Figures S18–S19) and displayed on a smartphone interface via Bluetooth communication. In a representative participant, the initial 10 min were treated as a warm‐up period associated with sweat accumulation and wetting of the sensing region. Thereafter, the glucose signal gradually stabilized at approximately 56.7 ± 0.47 µM. Similar trends are observed in the other two participants, with final concentrations of 57.0 ± 0.82 µM and 62.3 ± 1.25 µM, respectively, suggesting that sweat glucose monitoring reflects individual metabolic states and may aid in early metabolic risks (Figure 5f–i). To further evaluate the analytical accuracy of the wearable platform, sweat samples were collected from three participants at 10, 20, and 30 min during exercise and analyzed by HPLC. The corresponding sweat patch measurements were paired with the HPLC‐derived glucose concentrations, yielding nine paired observations. Bland–Altman analysis demonstrated reasonable agreement between the two methods, with a mean bias of −1.53 µM and 95% limits of agreement from −6.96 to 3.89 µM (Figure 5j). These results support the feasibility of the wearable sensor for quantitative sweat glucose monitoring within the investigated concentration range. The successful on‐body demonstration highlights the reliability of the wearable sweat patch and its potential for personalized metabolic tracking. By enabling continuous, enzyme‐free sweat glucose monitoring, the system supports proactive health management and holds promise for applications in fitness tracking, longitudinal metabolic monitoring, and chronic disease follow‐up.

### 2.5 Glucose Level Correlation Analysis Across Multiple Biofluids

Glucose metabolism involves a sequential physiological pathway from dietary intake to systemic distribution across interconnected biofluid compartments (Figure 6a). Carbohydrates consumed in food are hydrolyzed into polysaccharides and subsequently into monosaccharides, primarily glucose, which are absorbed into the bloodstream, diffuse into the ISF, and eventually appear in sweat through eccrine secretion [51]. Leveraging the high‐performance Au─Ni(OH)_2_‐based non‐enzymatic glucose sensor, we achieved stable in situ sweat glucose monitoring using the epidermal patch. To enable comprehensive physiological correlation analysis, glucose concentrations in ISF and blood were simultaneously measured using commercial clinical instruments and directly compared with sweat glucose values (Figure S20). Integrating these datasets reveals the physiological relevance and quantitative correlations of glucose levels across sweat, ISF, and blood levels under real‐world conditions.

**FIGURE 6:**
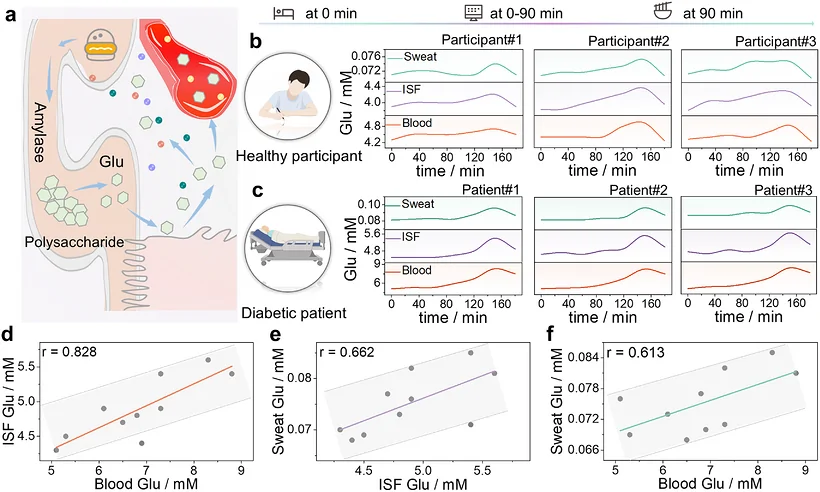
Correlation analysis of glucose in sweat, ISF, and blood. (a) Schematic illustration of the glucose metabolism pathway in the human body. (b, c) Dynamic changes in sweat, ISF, and blood glucose levels during daily activities in healthy participants (b) and diabetic patients (c), respectively (n = 3). (d–f) Pearson correlation analysis based on temporally matched endpoint glucose measurements from healthy participants (n = 5) and diabetic patients (n = 5; total n = 10): (d) blood *vs*. ISF (*p* = 0.003), (e) ISF *vs*. sweat (*p* = 0.037), and (f) blood *vs*. sweat (*p* = 0.059).

To evaluate glucose dynamics under daily‐life conditions, a separate 3‐h continuous monitoring experiment was performed from 10:00 to 13:00 (Figure 6b,c and Figure S21). The detailed information regarding participant characteristics have been provided in Table S2. Glucose levels in sweat, ISF, and blood were recorded at 20‐min intervals. Monitoring was initiated at 10:00 under preprandial conditions, and the participants consumed lunch at 11:30. Measurements were then continued until 13:00 to capture the postprandial glucose response. During the preprandial period, glucose levels in the three biofluids remained relatively stable. Following food intake, glucose levels in blood, ISF, and sweat increased with different amplitudes and temporal profiles and subsequently tended toward recovery. These dynamic changes reflect the physiological processes of glucose absorption, systemic distribution, and metabolic regulation. In the healthy individual, sweat glucose increases from 71.3 ± 0.47 µM to 74.0 ± 0.82 µM (4% change), ISF from 4.00 ± 0.08 mM to 4.27 ± 0.05 mM (7% change), and blood glucose from 4.47 ± 0.05 mM to 4.83 ± 0.12 mM (8% change). In contrast, the diabetic participant experiences markedly amplified postprandial responses, with sweat glucose rising from 82.0 ± 0.82 µM to 96.3 ± 0.47 µM (17%), ISF glucose from 4.43 ± 0.05 mM to 5.50 ± 0.08 mM (24%), and blood glucose from 5.27 ± 0.05 mM to 8.40 ± 0.08 mM (59%). These observations indicate larger postprandial glucose excursions in the monitored diabetic participants.

In a separate cross‐biofluid correlation experiment, five healthy and five diabetic participants were evaluated using a standardized postprandial protocol. Statistical analysis across all ten participants reveals positive cross‐biofluid associations among the three biofluids (Figure 6d–f). Pearson correlation coefficients are 0.828 between blood and ISF, 0.662 between ISF and sweat, and 0.613 between blood and sweat, confirming that sweat glucose is physiologically linked to systemic glucose dynamics. Furthermore, to better illustrate interindividual variability, exploratory subgroup analyses were also performed for healthy and diabetic participants (Figures S22 and S23). In healthy participants, the Pearson correlation coefficients are 0.691 between blood and ISF, 0.273 between ISF and sweat, and −0.434 between blood and sweat. In diabetic participants, the Pearson correlation coefficients are 0.847 between blood and ISF, 0.663 between ISF and sweat, and 0.816 between blood and sweat. We would like to emphasize that these subgroup analyses are exploratory due to the limited number of participants in each group. Instead, they illustrate subgroup‐level variability and the exploratory nature of the observed cross‐biofluid associations of sweat glucose changes across different populations, underscoring the potential of sweat‐based sensing for personalized metabolic monitoring and risk assessment. Physiological transport from blood to ISF and subsequently to sweat may introduce biofluid‐specific temporal delays, which can attenuate correlations calculated at matched clock times and contribute to interindividual variability. Accordingly, the Pearson analysis was performed using temporally matched measurements without post hoc lag correction, and the resulting coefficients should be interpreted as zero‐lag physiological associations rather than instantaneous equivalence among biofluids. Resolving individualized lag dynamics with higher temporal resolution will be important for further translating sweat glucose from a wearable analytical signal into a quantitatively interpretable systemic biomarker.

Collectively, these results establish a quantitative and dynamic relationship among blood, ISF, and sweat glucose, bridging the gap between non‐invasive monitoring and clinically relevant biomarkers. They demonstrate the capability of our non‐enzymatic epidermal sweat patch to provide reliable, real‐time metabolic assessment and potential metabolic risk assessment in both healthy and at‐risk populations. By integrating physiological correlation analysis with wearable biosensing, this work lays a foundation for the clinical translation of sweat‐based diagnostics and supports the broader adoption of non‐invasive health monitoring technologies.

## 3 Conclusion

To overcome the limitations of current sweat‐sensing technologies and the poorly defined relationships between sweat and other biofluids, we developed an epidermal sweat patch capable of in situ, continuous, and skin‐integrated glucose monitoring. The device leverages Au‐Ni(OH)_2_ aerogels as the sensing material, achieving high sensitivity and stability toward glucose owing to enhanced reactant adsorption by Ni(OH)_2_ and improved thermodynamic favorability at exposed Au active sites, as supported by electrochemical characterization and DFT calculations. Integrated with a microfluidic module for efficient sweat collection and onboard electronics for signal processing and wireless transmission, the patch enables real‐time, on‐body tracking of glucose dynamics. Human studies further reveal positive cross‐biofluid associations among sweat, ISF, and blood glucose, as well as characteristic fluctuation patterns across daily activities, highlighting the potential of sweat‐based sensing as a viable alternative for metabolic surveillance. Overall, this work establishes a practical route toward non‐invasive glucose monitoring and lays the foundation for broader applications in metabolic health assessment and chronic disease management.

## 4 Experimental Section

### 4.1 Synthesis of Au‐Ni(OH)_2_ Dual Aerogels

Au‐Ni(OH)_2_ dual aerogels were synthesized via a NaBH_4_‐induced one‐step gelation process, where pH regulation of the precursor solution is critical. Briefly, 25.6 µL of HAuCl_4_∙3H_2_O (10 wt.%) and 67 µL of NiCl_2_·6H_2_O (0.1 M) were added to 50 mL of ultrapure water and thoroughly stirred. The solution pH was adjusted to 10 using freshly prepared NaOH solution (4 M), followed by rapid injection of freshly prepared NaBH_4_ solution (7 mg mL^−1^). Within 15 min, the solution color transitioned from wine red to gray and then black, after which stirring was stopped. The mixture was left undisturbed overnight to form black, jelly‐like Au‐Ni(OH)_2_ hydrogels was formed, which was washed with water (10 times) and freeze‐dried to obtain the aerogels. For comparison, Ni(OH)_2_ and Au aerogels were synthesized using a similar procedure by substituting the corresponding metal precursors.

### 4.2 HPLC Assay for Product Analysis

Glucose oxidation products generated during electrolysis were analyzed via HPLC equipped with a refractive index detector. After long‐term electrochemical testing, 0.1 mL of the electrolyte was collected from the electrochemical cell and diluted with 1.0 M phosphate (H_3_PO_4_, 0.5 mL) solution to adjust pH<7.0. For the reactant, 5 mM H_2_SO_4_ was used as the mobile phase at a flow rate of 0.5 mL min^−1^. A 10 µL aliquot of the diluted sample was injected into a Bio‐Rad Aminex HPX‐87H column maintained at 35°C. For gluconic acid analysis, dipotassium hydrogen phosphate solution served as the mobile phase at a flow rate of 0.7 mL min^−1^. A 10 µL aliquot of the diluted sample was injected into a Kromasil C18 column, also maintained at 35°C.

### 4.3 Preparation of Glucose Sensing Electrodes

The non‐enzymatic glucose sensing was achieved by direct electrocatalysis of Au‐Ni(OH)_2_ aerogels. Glassy carbon electrodes (GCEs) and screen‐printed electrodes (SPEs) were used throughout the experiments. GCEs were polished with 0.05 µm alumina and sequentially cleaned by ultrasonic treatment in ethanol and ultrapure water. SPEs were cleaned in the same manner, without polishing. A suspension of Au‐Ni(OH)_2_ dual aerogels (1 mg mL^−1^, 5 µL) was drop‐cast onto the GCEs/SPEs (referred to coated onto as Au‐Ni(OH)_2_/GCEs or Au‐Ni(OH)_2_/SPEs) and allowed to air‐dry under ambient conditions. Subsequently, 2% chitosan solution (3 µL) was coated onto the modified electrode surface and left to dry naturally. For comparison, Ni(OH)_2_/GCEs or SPEs and Au gel/GCEs or SPEs were prepared using the same procedure with the corresponding sensing materials.

### 4.4 Fabrication of the Microfluidic Chip

The microfluidic chip was fabricated using standard photolithography. SU‐8 photoresist was patterned on a silicon wafer according to designs generated in CAD. PDMS (base to curing agent ratio of 10:1) was poured onto the SU‐8 mold and degassed under vacuum to remove air bubbles, then cured at 60°C for 3 h. Inlets and outlets for sweat collection and evaporation were created using mechanical punches. The PDMS chip was bonded to human skin using a double‐sided adhesive layer with pre‐aligned holes.

### 4.5 Construction of the Epidermal Sweat Patch

The epidermal sweat patch comprised an Au‐Ni(OH)_2_ aerogel‐based SPE integrated with a microfluidic chip, electronic circuitry, and a wireless transmission module. In wearable applications, sweat collected by the microfluidic chip served as the electrolyte to wet the SPE, while the electronic circuitry transmitted sensing data to a smartphone interface via Bluetooth. The recorded signals were subsequently converted into glucose concentration values using pre‐established calibration curves, enabling in situ, real‐time monitoring of glucose levels in epidermal sweat.

### 4.6 On‐Body Analysis

The on‐body study was designed to evaluate the performance of the developed epidermal sweat patch for glucose monitoring and cross‐biofluid correlation analysis. For the daily‐activity monitoring, glucose levels in sweat, ISF, and blood were monitored over a 3‐h period from 10:00 to 13:00 at 20‐min intervals. Monitoring was initiated at 10:00 under preprandial conditions. Participants consumed lunch at 11:30, and measurements were continued until 13:00 to characterize the subsequent postprandial glucose dynamics. A separate standardized postprandial experiment was conducted for cross‐biofluid correlation analysis. Five healthy adults and five diabetic participants were recruited. To minimize interindividual variability associated with food composition, portion size, and meal timing, all participants were provided with an identical boxed lunch of the same portion size by the cafeteria of Xi'an Jiaotong University at 12:00, and each participant consumed the entire meal and completed eating at 12:30. At 13:00, corresponding to 30 min after completion of the meal, simultaneous monitoring of glucose levels in sweat, ISF, and blood was initiated.

Sweat glucose was monitored using the developed epidermal sweat patch, while ISF and blood glucose levels were measured using commercially available devices (FreeStyle Libre, Abbott, for ISF glucose; home blood glucose meter, Sinocare, for blood glucose). Measurements from the three biofluids were temporally matched according to the predefined monitoring schedule. For Pearson correlation analysis, the endpoint glucose values obtained at the final scheduled measurement were used, yielding one paired participant‐level observation from each participant and a total of n = 10 observations. All procedures involving human participants were approved by the Medical Ethics Committee of the Second Affiliated Hospital of Xi'an Jiaotong University (Approval No. 2025YS731). Written informed consent was obtained from all participants prior to enrollment in the study.

## Author Contributions

**Yao Chen**: conceptualization, data curation, investigation, writing – original draft. **Xinhao Wan**: data curation, software, investigation. **Haoxin Fan**: investigation. **Hengwei Lou**: investigation. **Jialu Wang**: investigation. **Jie Gao**: software. **Dan Wen**: supervision, funding acquisition, project administration, writing – review and editing.

## Conflicts of Interest

The authors declare no conflicts of interest.

## Supporting information




**Supporting File**: advs78544‐sup‐0001‐SuppMat.docx.

## Data Availability

The data that support the findings of this study are available from the corresponding author upon reasonable request.
