# Supplementary material for: An Epidermal Sweat Patch for Tracking Metabolite Levels and Correlation Analysis in Biofluids
Source: Adv Sci (Weinh). 2026 Oct 29:e78544. Online ahead of print. doi: 10.1002/advs.78544 (PMC13720386; doi:10.1002/advs.78544)
Supplement: Supplementary file 1 [file ADVS-9999-e78544-s001.docx]

Supporting Information

An Epidermal Sweat Patch for Tracking Metabolite Levels and Correlation Analysis in Biofluids

*Yao Chen^1,2^, Xinhao Wan^2^, Haoxin Fan^2^, Hengwei Lou^2^, Jialu Wang^2^, Jie Gao^3^ and Dan Wen^2*^*

^1^Department of Hepatobiliary Surgery and Liver Transplantation, The Second Affiliated Hospital of Xi’an Jiaotong University, Xi’an, 710004, China

^2^State Key Laboratory of Solidification Processing, School of Materials Science and Engineering, Northwestern Polytechnical University, Xi’an, 710072, P. R. China

^3^School of Life Sciences, Northwestern Polytechnical University, Xi’an, 710072, P. R. China

^*^Corresponding author: D. Wen ([dan.wen@nwpu.edu.cn](mailto:dan.wen@nwpu.edu.cn)).

**Experimental Section**

**Reagents and Materials**

Gold (III) chloride trihydrate (HAuCl_4_∙3H_2_O, 99.9%), nickel (Ⅱ) chloride hexahydrate (NiCl_2_∙6H_2_O, 99.9%), sodium borohydride (NaBH_4_), sodium hydroxide (NaOH), glucose, lactate, urea, sodium chloride (NaCl, 99.9%), potassium chloride (KCl, 99.9%), calcium chloride (CaCl_2_, 99.9%), uric acid (UA) and ascorbic acid (AA) were all bought from Sigma-Aldrich Co., Ltd (USA). Sodium dihydrogen phosphate (NaH_2_PO_4_, 99.9%), disodium hydrogen phosphate (Na_2_HPO_4_, 99.9%), gluconic acid and polydimethylsiloxane (PDMS) were obtained from Shanghai Sinopharm Chemical Reagent Co., Ltd. Artificial sweat (pH = 6.5) and artificial interstitial fluid (ISF, pH = 7.4) were prepared according to our previous work^[1]^. All chemicals were of analytical grade, and all solutions were freshly prepared using ultrapure water (18.2 MΩ·cm).

**Apparatus and Measurements**

Scanning electron microscopy (SEM) images were obtained from a FEI nova nanoSEM 450 scanning electron microscope with an accelerating voltage of 15 kV. Transmission electron microscopy (TEM) images, high-resolution TEM (HRTEM) images, elemental mapping, high-angle annular dark-field scanning transmission electron microscopy (HAADF-STEM) images, and selected area electron diffraction (SAED) patterns were acquired from a Tecnai G2 F30 transmission electron microscope at 200 kV. X-ray photoelectron spectroscopy (XPS) measurements were performed using a photoelectron spectrometer (ESCALAB 250XI, Thermo Fisher). The C 1s peak (284.8 eV) was selected as a reference to calibrate binding energies, and Al Kα X-ray radiation was used as the X-ray source. Powder X-ray diffraction (XRD) was carried out in reflection mode on a X’Pert Pro multipurpose powder diffractometer operated at a voltage of 40 kV and a current of 30 mA with Cu Kα radiation (λ = 1.5406 Å). The solution pH value was measured using a commercial pH meter (Inesa, PHS-3E). In situ Fourier transform infrared (FT-IR) spectra were recorded on a Nicolet iS50 FTIR spectrometer (Thermo Scientific, USA). High-performance liquid chromatography (HPLC) was performed on an Agilent 1290 Infinity II system.

All the electrochemical measurements were conducted on a CHI 842D electrochemical workstation (Shanghai Chenhua Instrument Corporation, China), coupled with a conventional three-electrode system. Cyclic voltammetry (CV) measurements were carried out in the range of -0.3–0.7 V at a scan rate of 50 mV s^−1^ unless otherwise specified. Amperometric i-t curves were recorded at a constant potential of +0.1 V. Open-circuit potential measurements were performed with a sample interval of 0.1 s, over a potential range of -0.5–0.5 V.

**HPLC Validation of Sweat Glucose Measurements**

Sweat samples for HPLC validation were collected during the on-body exercise experiments. Three participants were included in the validation study. For each participant, sweat samples were collected at three predefined time points, i.e., 10, 20, and 30 min after the start of exercise, yielding a total of nine paired sweat measurements.

At each sampling time point, the glucose concentration recorded by the wearable sweat sensor was documented, and a corresponding sweat sample was simultaneously collected for HPLC analysis. Glucose concentrations in the collected sweat samples were quantified using an Agilent 1290 Infinity II HPLC system equipped with a refractive-index detector. Chromatographic separation was performed using a Bio-Rad Aminex HPX-87H column maintained at 35 °C. A 5 mM H_2_SO_4_ solution was used as the mobile phase at a flow rate of 0.5 mL min^–1^, and a 10 μL aliquot of each sweat sample was injected for analysis. Glucose was identified by comparison of its retention time with that of an authentic glucose standard and quantified using an external calibration curve established with standard glucose solutions.

For method comparison, each HPLC-derived glucose concentration was paired with the wearable-sensor measurement obtained at the corresponding sampling time point. Agreement between the two methods was assessed using Bland–Altman analysis, with the difference between the wearable-sensor and HPLC measurements plotted against the mean of the paired measurements.

**Numerical Simulation**

Numerical simulations were conducted using the finite-element software COMSOL Multiphysics 5.6 (COMSOL Co., Ltd., Beijing, China). The microfluidic chip model was first designed in AutoCAD. The velocity profile within the microfluidic chamber at different flow rates was then simulated by numerically solving the Stokes equations for incompressible flow using COMSOL Multiphysics.

**Density Functional Theory (DFT) Calculations**

DFT calculations were performed using the Vienna Ab initio Simulation Package (VASP)^[2, 3]^. The interactions between ion cores and valence electrons were calculated with the projector augmented wave (PAW) method^[4]^. The exchanged-correlation interactions were handled by use of a generalized gradient approximation with the Perdew-Burke-Ernzerhof (PBE) function^[5]^. Valence electronic states were expanded in a plane-wave basis set with a cutoff energy of 450 eV. Convergence criteria for total energy and force were set to 1 × 10^-5^ eV and -0.02 eV·Å^-1^, respectively. A 3×3×1 grid was adopted in the Brillouin zone which was modeled by gamma centered Monkhorst-Pack scheme.

For the computational study of glucose oxidation, the Grimme DFT-D3 method was employed to correct for van der Waals interactions, thereby enhancing calculation accuracy. A four-layer face-centered cubic (4×4×4) extended structure of the Au(111) lattice was constructed, with the bottom two atomic layers fixed in position to improve computational efficiency. Additionally, a bilayer Ni(OH)_2_ model with a 5×5 supercell was established, and a 19-atom gold cluster was deposited above the Ni(OH)_2_ layer to form the Au-Ni(OH)_2_ composite system. To eliminate periodic boundary interactions, a 15 Å vacuum layer was incorporated to isolate the system.

We calculated the free energy change by the following formula:

$\Delta G=\Delta E+\Delta ZPE+\Delta H-T\Delta S+\Delta G_{\mathrm{pH}}+\Delta G_{u}$	(1)

where ΔE, ΔZPE, ΔH and the ΔS were referred to as the change in potential energy, the change in the zero-point energy, the integrated heat capacity from 0 K to 298.15 K, and the change in the entropy. $\Delta G_{\mathrm{pH}}=-K_{B}T\ln H^{+}=pH\times K_{B}\mathrm{Tln} 10$, where K_B_ is the Boltzmann constant. $\Delta G_{u}=-neU$, U represents the applied electrode potential, n is the number of proton-electron pairs transferred and e is the charge transferred, respectively.

The zero-point energy was calculated by the summation of all vibrational frequencies:

$ZPE=\frac{1}{2}\sum h\nu$	(2)

where the ν corresponded to the vibrational frequency of each normal mode.

During the DFT calculation, the adsorption energies for intermediates at 298 K were calculated in the following way:

$E_{\mathbf{Glu}_{\mathbf{ads}}}={E*}_{\mathbf{Glu}}-E_{\mathbf{Glu}}-E*$	(3)

**Supporting Figures**

**Figure S1.** The line scanning curves (a) and energy dispersive spectroscopy (b) of Au-Ni(OH)_2_ aerogels.

**Figure S2.** The survey XPS spectrum of the Au-Ni(OH)_2_ aerogels.

**Figure S3.** (a, b) CV curves of Ni(OH)_2_ and Au gel-modified electrodes for glucose oxidation in 0.1 M phosphate-buffered saline (PBS) (pH 7.4).

**Figure S4.** (a) CVs of different aerogels-modified glassy carbon electrodes (GCEs) in 0.1 M KCl solution containing 5 mM [Fe(CN)_6_]^3−/4−^; (b) Histograms of the electrochemical active surface areas for the modified electrodes.

**Figure S5.** The geometric configuration of the adsorbed glucose on Au(111) (a), Ni(OH)_2_ (b) and Au-Ni(OH)_2_ (c), respectively.

**Figure S6.** I-t curves (a) and the corresponding comparison of the current response (b) at the different applied potential.

**Figure S7.** Effects of pH and temperature on the glucose-sensing response of the Au–Ni(OH)_2_-based sensor. Normalized current responses measured at different pH values (5.0–7.4) (a) and at different temperatures (25–40 °C) (b) in the presence of 1 mM glucose.

**Figure S8.** I-t curves (a) and the corresponding calibration plot (b) of Au-Ni(OH)_2_/screen-printed electrode (SPE) in artificial sweat (pH 6.5) at an applied potential of 0.1 V (*vs* Ag/AgCl). (c) Normalized current of the sensor in response to 50 μM AA and other interferents (0.05-5 mM). (d) Histograms of the current response of the different SPEs.

**Figure S9.** I-t curves (a) and the corresponding calibration plot (b) of Au-Ni(OH)_2_/SPE in artificial ISF (pH 7.4) at an applied potential of 0.1 V (*vs* Ag/AgCl).

**Figure S10.** I-t curves (a) and the corresponding calibration plot (b) of Au-Ni(OH)_2_/SPE in human serum (pH 7.4) at an applied potential of 0.1 V (*vs* Ag/AgCl).

**Figure S11.** Schematic design and geometric parameters of the microfluidic chip.

The width and height of the internal microfluidic channels are 200 and 200 μm, respectively. The inlet and outlet diameters are both 2 mm, whereas the sensing reservoir has a diameter of 6 mm.

**Figure S12.** Fabrication process of the microfluidic chip.

**Figure S13.** (a−c) Optical images of the microfluidic chip.

The flexible microfluidic chip can conformally adhere to human skin and shows good flexibility, indicating its potential for wearable applications in various physical activities.

**Figure S14.** Schematic illustration of the sweat flow in the microfluidic chip operated on the skin in cross-sectional views.

An outlet with the same size as the inlets is adopted to avoid backpressure. Sweat secreted from the sweat glands enters and fills the sensing reservoir under glandular secretory pressure, thereby initiating glucose analysis. The sweat then flows toward the outlet and evaporates, maintaining unidirectional flow and minimizing mixing between newly secreted and previously collected sweat

**Figure S15.** Images of the theoretical simulation of the microfluidic chip for sweat collection at sweat flow rate of 0.2 µL min^−1^ (a) and 2 µL min^−1^ (b), respectively.

**Figure S16.** (a) Time dependence of the sweat collection volume of the microfluidic chip. The relationship between response signal and sweat collection volume (b) and time evolution at different sweat flow rates (c), respectively.

The collected sweat volume gradually increases and eventually becomes saturated (up to 8 μL) over time, allowing for highly stable biosensing in the sweat matrix. To validate the sensing performance of the microfluidic chip, dynamic biosensing was performed. The collected sweat volume is found to be linearly correlated with the response current. There are no significant changes observed in response signal at varying physiologically relevant sweat rates (0.2, 2 and 4 μL min^‒1^), only the response time varies. Specifically, at higher flow rates, the signal response is detected more quickly. Taken together, the as-designed microfluidic chip offers an attractive sweat collection and low sample volume quantitative analysis solution, suitable for low-volume sweat analysis.

**Figure S17.** I-t curves caused by different sweat sample volumes.

**Figure S18.** The overall design diagram of the device circuit.

**Figure S19.** The calibration curve between the glucose concentration in artificial sweat and the potential responses.

**Figure S20.** Optical photos showing glucose measurements in sweat, ISF, and blood.

**Figure S21.** Radar plots of sweat, ISF and blood glucose levels during daily activities in healthy participants (a-b) and diabetic patients (c-d), respectively.

**Figure S22.** Pearson correlation analysis in healthy participants (n=5): (a) sweat vs. ISF (p=0.656), (b) sweat vs. blood (p=0.465), and (c) ISF vs. blood (p=0.197).

**Figure S23.** Pearson correlation analysis in diabetic patients (n=5): (a) sweat vs. ISF (p=0.223), (b) sweat vs. blood (p=0.092), and (c) ISF vs. blood (p=0.07).

**Table S1.** Comparison of the limit of detection (LOD) and stability of the glucose sensor with previously reported glucose sensing platforms.

| **Electrode materials** | **Stability/days** | **LOD/µM** | **Wearable sensor** | **Ref.** |
| --- | --- | --- | --- | --- |
| Au-Ni(OH)_2_ | 35 | 0.1 | Yes | This work |
| rGO/PU | 7 | 0.5 | Yes | [6] |
| Pt_1_/Ni(OH)_2_ | 28 | 18 | No | [7] |
| Au_200_Bi | 15 | 8.7 | No | [8] |
| Ru_1_Cu | 30 | 5.0 | No | [9] |
| single-atom Ir | 4.17 | 0.67 | No | [10] |
| Ni SAs/graphene | 0.046 | 0.19 | No | [11] |
| Pt_1_/Ni_6_Co_1_ | 35 | 19 | No | [12] |
| Au NPs/Ni-BDC | 15 | 6.29 | Yes | [13] |
| Alginate hydrogel | 30 | 10.4 | No | [14] |
| Pt NPs | 14 | 236.42 | Yes | [15] |
| Aptamer | 3 | 1.04 | Yes | [16] |
| AgNPs | 0.057 | 80 | No | [17] |
| boron carbide | 28 | 35 | Yes | [18] |
| MXene-GO | / | 106.8 | Yes | [19] |

**Table S2.** Clinical characteristics of study participants.

| **Participants** | **Age** | **Gender** | **Physical examination** | | **Diabetes type** | **Group** |
| --- | --- | --- | --- | --- | --- | --- |
|  |  |  | **Height/cm** | **Weight/kg** |  |  |
| 1 | 23 | Male | 176 | 74 | / | Healthy participant |
| 2 | 28 | Female | 162 | 55 | / |  |
| 3 | 31 | Female | 170 | 50 | / |  |
| 4 | 30 | Male | 181 | 84 | / |  |
| 5 | 26 | Male | 163 | 53 | / |  |
| 6 | 57 | Female | 158 | 43 | Type 2 | Diabetes |
| 7 | 55 | Male | 173 | 65 | Type 2 |  |
| 8 | 42 | Female | 160 | 54 | Type 2 |  |
| 9 | 39 | Female | 164 | 49 | Type 2 |  |
| 10 | 34 | Male | 175 | 75 | Type 2 |  |

**References**

[1] Y. Chen, X. Wan, G. Li, et al., “Metal Hydrogel-Based Integrated Wearable Biofuel Cell for Self-Powered Epidermal Sweat Biomarker Monitoring,” *Advanced Functional Materials* 34 (2024): 2404329.
[2] G. Kresse and J. Furthmüller, “Efficiency of Ab-Initio Total Energy Calculations for Metals and Semiconductors Using a Plane-Wave Basis Set,” *Computational Materials Science* 6, (1996): 15–50.
[3] L. C. Grabow, A. A. Gokhale, S. T. Evans, et al., “Mechanism of the Water Gas Shift Reaction on Pt: First Principles, Experiments, and Microkinetic Modeling,” *The Journal of Physical Chemistry C* 112, 12 (2008): 4608–4617.
[4] P. E. Blöchl, “Projector Augmented-Wave Method,” *Physical Review B* 50, 24 (1994): 17953–17979.
[5] J. P. Perdew, K. Burke, and M. Ernzerhof, “Generalized Gradient Approximation Made Simple,” *Physical Review Letters* 77, 18 (1996): 3865–3868.
[6] P. T. Toi, T. Q. Trung, T. M. L. Dang, et al., “Highly Electrocatalytic, Durable, and Stretchable Nanohybrid Fiber for On-Body Sweat Glucose Detection,” *ACS Applied Materials & Interfaces* 11 (2019): 10707–10717.
[7] B. Long, Y. Zhao, P. Cao, et al., “Single-Atom Pt Boosting Electrochemical Nonenzymatic Glucose Sensing on Ni(OH)_2_/N-Doped Graphene,” *Analytical Chemistry* 94 (2022): 1919–1924.
[8] Q. Fang, Y. Qin, H. Wang, et al., “Ultra-Low Content Bismuth-Anchored Gold Aerogels with Plasmon Property for Enhanced Nonenzymatic Electrochemical Glucose Sensing,” *Analytical Chemistry* 94 (2022): 11030–11037.
[9] S. Zhang, Y. Jiang, W. Lei, et al., “Tailoring the d-Band Center on Ru_1_Cu Single-Atom Alloy Nanotubes for Boosting Electrochemical Non-Enzymatic Glucose Sensing,” *Analytical and Bioanalytical Chemistry* 416 (2024): 6103–6111.
[10] X. Luo, M. Yang, W. Song, et al., “Neutral Zn-Air Battery Assembled with Single-Atom Iridium Catalysts for Sensitive Self-Powered Sensing System,” *Advanced Functional Materials* 31 (2021): 2101193.
[11] H. Yin, X. Bai, F. Zhang, et al., “Dual Single Atomic Ni Sites Constructing Janus Hollow Graphene for Boosting Electrochemical Sensing of Glucose,” *Microchimica Acta* 191 (2024): 314.
[12] B. Long, P. Cao, Y. Zhao, et al., “Pt_1_/Ni_6_Co_1_ Layered Double Hydroxides/N-Doped Graphene for Electrochemical Non-Enzymatic Glucose Sensing by Synergistic Enhancement of Single Atoms and Doping,” *Nano Research* 16 (2023): 318–324.
[13] Y. Yang, W. Huang, X. Lei, et al., “Wearable Wireless Patch with Unidirectional Microfluidic Chamber for Enzyme-Free Glucose Monitoring in Sweat,” *ACS Sensors* 10 (2025): 4401–4411.
[14] V. Marchianò, C. Pellegrini, A. Tricase, et al., “Mussel-Bioinspired Edible Ca^2+^-Crosslinked Alginate Hydrogel Electrodes for Glucose Gastrointestinal Monitoring,” *Advanced Science* 12 (2025): e16912.
[15] W. Zhu, H. Yu, W. Li, et al., “pH Calibration Allows Accurate Glucose Detection in Interstitial Fluid *via* Reverse Iontophoresis,” *Nature Communications* 16 (2025): 10413.
[16] F. Bakhshandeh, H. Zheng, N. G. Barra, et al., “Wearable Aptalyzer Integrates Microneedle and Electrochemical Sensing for In Vivo Monitoring of Glucose and Lactate in Live Animals,” *Advanced Materials* 36 (2024): 2313743.
[17] X. Wang, H. Huo, Y. Zhong, et al., “Synergistic Antimicrobial Glycyrrhizic Acid-Based Functional Biosensing Composite for Sensitive Glucose Monitoring and Collaborative Wound Healing,” *Advanced Healthcare Materials* 13 (2024): 2400580.
[18] Z. Wang, X. Wang, H. Wen, et al., “Boron Carbide Nanoskeleton-Engineered Wearable Biosensor for Real-Time Sweat Glucose Monitoring,” *Advanced Healthcare Materials* 14 (2025): e03624.
[19] R. Li, H. Fan, Y. Chen, et al., “MXene-Graphene Oxide Heterostructured Films for Enhanced Metasurface Plasmonic Biosensing in Continuous Glucose Monitoring,” *Advanced Science* 12 (2025): 2410376.
